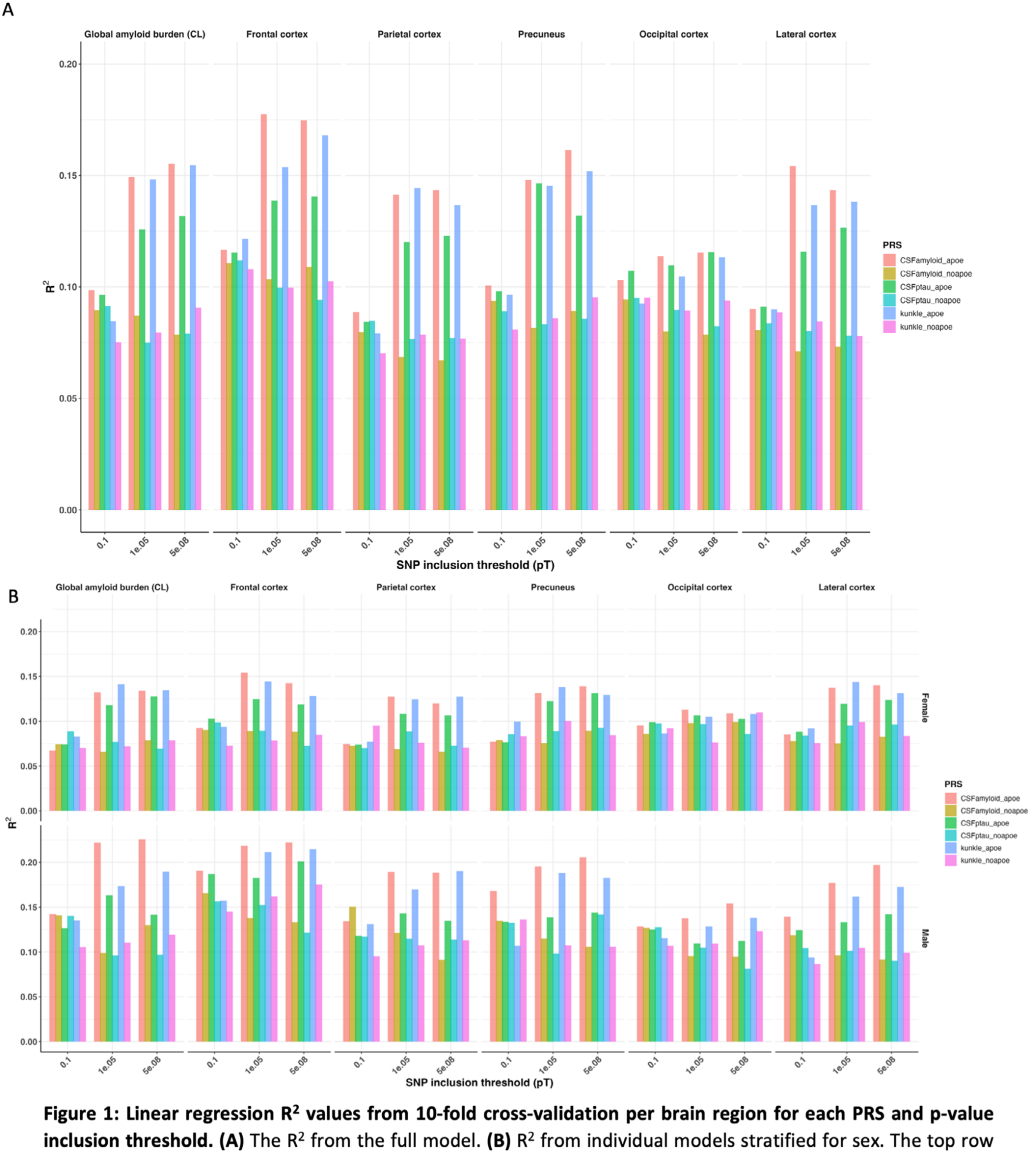# Associations between regional amyloid PET and genome‐wide polygenic risk scores in the predementia AMYPAD consortium

**DOI:** 10.1002/alz70855_104356

**Published:** 2025-12-24

**Authors:** Emma S. Luckett, Yasmina Abakkouy, Luigi Lorenzini, Lyduine E. Collij, Natalia Vilor‐Tejedor, Frederik Barkhof, Rik Vandenberghe, Isabelle Cleynen, AMYPAD consortium

**Affiliations:** ^1^ Amsterdam UMC location VUmc, Amsterdam, Netherlands; ^2^ Laboratory for Complex Genetics, KU Leuven, Leuven, Belgium; ^3^ Laboratory for Cognitive Neurology, Leuven Brain Institute, KU Leuven, Leuven, Belgium; ^4^ Amsterdam Neuroscience, Brain Imaging, Amsterdam, Netherlands; ^5^ Amsterdam UMC location VUmc, Amsterdam, Amsterdam, Netherlands; ^6^ Clinical Memory Research Unit, Department of Clinical Sciences Malmö, Faculty of Medicine, Lund University, Lund, Sweden; ^7^ Hospital del Mar Research Institute, Barcelona, Spain; ^8^ Barcelonaβeta Brain Research Center (BBRC), Pasqual Maragall Foundation, Barcelona, Spain; ^9^ Center for Genomic Regulation, Barcelona, Spain; ^10^ Radboud University Medical Center, Nijmegen, Netherlands; ^11^ Queen Square Institute of Neurology and Centre for Medical Image Computing, University College London, London, Greater London, United Kingdom; ^12^ Amsterdam UMC, location VUmc, Amsterdam, Noord‐Holland, Netherlands; ^13^ Department of Radiology and Nuclear Medicine, Vrije Universiteit Amsterdam, Amsterdam University Medical Center, location VUmc, Amsterdam, Netherlands; ^14^ Laboratory for Cognitive Neurology, KU Leuven, Leuven, Leuven, Belgium; ^15^ Alzheimer Research Centre KU Leuven, Leuven Brain Institute, Leuven, Belgium; ^16^ The project leading to this paper has received funding from the Innovative Medicines Initiative 2 Joint Undertaking under grant agreement No 115952, Brussels, Belgium

## Abstract

**Background:**

Variability of brain amyloid burden across individuals suggests that biological factors may play a role. This study investigates the association between polygenic risk scores (PRS) and amyloid burden in a preclinical Alzheimer's disease (AD) cohort to identify genetic determinants.

**Method:**

PRS for AD susceptibility (Kunkle et al., 2019) and for CSF‐Aβ42 and ptau181 (Jansen et al., 2022) were computed for 867 non‐demented AMYPAD participants. PRS were calculated with and without the *APOE* region (19:44.4‐45.5Mb) under three *p*‐value inclusion thresholds (pT=5x10⁻⁸, 1x10⁻⁵, 0.1). Linear regression models tested associations with amyloid burden (Centiloid) adjusting for age, sex, and education (years). Model performance was evaluated using 10‐fold cross‐validation (R^2^). SNPs from the top‐performing PRS for each brain region were mapped to genes using biomaRt, followed by enrichment analysis with enrichGO. Separate linear regressions for males and females assessed sex differences.

**Result:**

Participants were predominantly cognitively unimpaired (CDR=0, 85.1%), with low amyloid burden (CL<10, 60%), and 42.4% carried at least one *APOE‐ε4* allele. The strongest predictive ability was observed for PRS_amyloid_ at pT=1x10⁻⁵ for frontal amyloid burden (R^2^=0.18, Figure 1A). PRS_amyloid_ and PRS_Kunkle_ at pT=5x10^‐8^ and 1x10^‐5^ performed comparably across brain regions (R^2^>0.14). At pT=1x10^‐5^, PRS_amyloid_ had 23 of 38 SNPs mapped to genes, enriched for acylglycerol (*p* = 0.006) and triglyceride (*p* = 0.005) homeostasis. PRS_Kunkle_ had 50 of 99 SNPs mapped to genes, enriched for negative‐regulation‐of‐amyloid‐beta‐formation (*p* = 1.9x10^‐6^) and amyloid‐precursor‐protein‐catabolic‐process (*p* = 2.8x10^‐6^). Sixteen mapped genes overlapped between the two scores. PRS at pT=0.1 consistently performed worse (R^2^<0.12), as did PRS_noAPOE_ (R^2^<0.11). Stratification by sex showed higher R^2^ for males (*N* = 354) than females (*N* = 512), with similar observations as above: PRS_amyloid_ and PRS_Kunkle_ at pT=5x10^‐8^ and 1x10^‐5^ showed comparable performance (Figure 1B). Overall, predictive performance was highest in the frontal cortex, comparable with the global composite in men, and lowest in the occipital cortex.

**Conclusion:**

PRS prediction was strongest in the frontal cortex, with PRS_amyloid_ and PRS_Kunkle_ performing similarly at stringent SNP inclusion thresholds. PRS_amyloid_ performed slightly better despite different biological enrichment. These findings, stronger in men, enhance our understanding of the genetic basis of amyloid pathology and support the potential of genetic profiling for identifying at‐risk individuals, with potential sex differences.